# A Critical Evaluation of Bifidobacterial Adhesion to the Host Tissue

**DOI:** 10.3389/fmicb.2016.01220

**Published:** 2016-08-05

**Authors:** Christina Westermann, Marita Gleinser, Sinéad C. Corr, Christian U. Riedel

**Affiliations:** ^1^Institute of Microbiology and Biotechnology, University of UlmUlm, Germany; ^2^Department of Microbiology, Moyne Institute of Preventative Medicine, School of Genetics and Microbiology, Trinity College DublinDublin, Ireland

**Keywords:** *Bifidobacterium*, probiotic, adhesion, host interaction

## Abstract

Bifidobacteria are common inhabitants of the human gastrointestinal tract that, despite a long history of research, have not shown any pathogenic potential whatsoever. By contrast, some bifidobacteria are associated with a number of health-related benefits for the host. The reported beneficial effects of bifidobacteria include competitive exclusion of pathogens, alleviation of symptoms of irritable bowel syndrome and inflammatory bowel disease, and modulation of intestinal and systemic immune responses. Based on these effects, bifidobacteria are widely used as probiotics by pharmaceutical and dairy industries. In order to exert a beneficial effect bifidobacteria have to, at least transiently, colonize the host in a sufficient population size. Besides other criteria such as resistance to manufacturing processes and intestinal transit, potential probiotic bacteria are tested for adhesion to the host structures including intestinal epithelial cells, mucus, and extracellular matrix components. In the present review article, we summarize the current knowledge on bifidobacterial structures that mediate adhesion to host tissue and compare these to similar structures of pathogenic bacteria. This reveals that most of the adhesive structures and mechanisms involved in adhesion of bifidobacteria to host tissue are similar or even identical to those employed by pathogens to cause disease. It is thus reasonable to assume that these structures and mechanisms are equally important for commensal or probiotic bacteria and play a similar role in the beneficial effects exerted by bifidobacteria.

## Introduction

The mammalian GIT is home to an extremely complex and diverse microbial ecosystem consisting primarily of prokaryotes. This microbial community is collectively referred to as the gut microbiota and exerts a number of profound effects on host health (Marchesi et al., 2016). During the first period of life when newborns are exclusively breast-fed, members of the genus *Bifidobacterium* are one of the predominant bacterial groups of the microbiota in the lower GIT (Yatsunenko et al., 2012; Bäckhed et al., 2015; Walker et al., 2015). The major source for bifidobacteria is the intestinal microbiota of the mother to which the newborn is exposed during (vaginal) delivery (Grönlund et al., 2011; Matamoros et al., 2013). More recently, breast milk has also been shown to contain viable bifidobacteria (Fernández et al., 2013; Jost et al., 2015). Although their relative proportion decreases over time, bifidobacteria are still a subdominant group amongst intestinal bacteria of adult humans (Arumugam et al., 2011).

With the exception of *Bifidobacterium dentium*, which has been associated with dental caries (Ventura et al., 2009), bifidobacteria have to date not shown any pathogenic potential. By contrast, a number of health promoting effects have been attributed to the presence of bifidobacteria in the GIT including improvement of symptoms of irritable bowel syndrome, inflammatory bowel disease and infectious diarrhea, modulation of intestinal and systemic immune responses, and resistance against colonization by pathogens (Gareau et al., 2010; Buffie and Pamer, 2013). Of note, a very recent study links bifidobacteria in the gut microbiota to enhanced anti-tumor immune responses and support of checkpoint-inhibition cancer therapy using a monoclonal antibody (Sivan et al., 2015). Based on these findings bifidobacteria are widely used as probiotics, i.e., live microorganisms which when administered in adequate amounts confer a health benefit to the host (Holmes et al., 2012; Foligné et al., 2013).

Besides the health promoting effects, several criteria are applied during selection of a suitable probiotic candidate strain including stability during manufacturing processes, viability during gastrointestinal transit and functionality at the desired target site (Foligné et al., 2013). One of the classical selection criteria for potential probiotic bacteria is adhesion to mucus and/or IECs (Klaenhammer and Kullen, 1999; Tuomola et al., 2001; Papadimitriou et al., 2015).

It may be argued that adhesion is not important for probiotic functionality since probiotic bacteria do not have access to host tissue due to the thick mucus layer covering the (healthy) gut epithelium. However, a number of bifidobacteria were shown to adhere to mucus (He et al., 2001; Izquierdo et al., 2008) and utilize host-derived mucins as a substrate for growth (Tailford et al., 2015). Also, bifidobacteria are discussed as potential treatment options for conditions with an impaired mucus layer (Whelan and Quigley, 2013; Johansson, 2014) facilitating direct access of (bifido)bacteria to the epithelium. Moreover, various bacterial pathogens must overcome the mucosal barriers and gain access to the epithelial layer to cause disease. For example, pathogenic *Escherichia coli* strains and related organisms use pili, fimbriae, and/or intimin with its translocated intimin receptor for adhesion to epithelial cells (Niemann et al., 2004). Another example is the interaction of InlA of *Listeria monocytogenes* with E-cadherin on host epithelial cells which is crucially required for infection (Stavru et al., 2011). Only once adhesion of these pathogens to the epithelium has been achieved despite the presence of an intact mucus layer, progression to later stages of infection and disease are possible (Bhavsar et al., 2007).

On the other hand, a number of probiotic traits may be directly linked to adhesion to host structures. One of the proposed health benefits of bifidobacteria is resistance against colonization or infection by pathogens. This may involve a variety of adhesion-independent mechanisms such as competition for nutrients or production of antimicrobial compounds (Buffie and Pamer, 2013; Lawley and Walker, 2013). Nevertheless, adhesion to IECs, mucus and ECM components by commensal and probiotic bacteria may also directly block access of pathogens to these structures (Bernet et al., 1993; Collado et al., 2005; Candela et al., 2008; Serafini et al., 2013) either by competition for attachment sites or steric hinderance. Also, there are numerous reports of immunomodulatory effects of bifidobacteria *in vitro* and in animal models (Bermudez-Brito et al., 2012). All these effects crucially depend on interaction with (and thus adhesion to) epithelial cells, dendritic cells, monocytes, macrophages and or other immune cells. Finally, even if not directly implicated mechanistically, adhesion might contribute to beneficial effects by allowing initial colonization or prolonging persistence of (probiotic) bifidobacteria in the GIT.

## Factors for Adhesion of Bifidobacteria to Host Structures

A number of factors and structures involved in adhesion to IECs, ECM components, and mucus have been identified in bifidobacteria (**Table 1**). These studies have been performed almost exclusively in *in vitro* model systems.

**Table 1:** Adhesive structures identified in bifidobacteria.

Structure/protein/property	Species	Evidence and role	Reference
Type IVb (Tad) pili	*B. bifidum, B. breve, B. longum* subsp. *longum, B. adolescentis*	Genes expressed *in vitro* and *in vivo*, for *B. breve* UCC2003: pili detected *ex vivo* by transmission electron microscopy, required for efficient colonization of mice	O’Connell Motherway et al., 2011; Westermann et al., 2012; Zhurina et al., 2013; Bottacini et al., 2014; Duranti et al., 2014, 2015
Type IVa pili	*B. adolescentis*	Genes expressed *in vitro*, regulation by carbon source	Duranti et al., 2014
Sortase-dependent pili	*B. adolescentis, B. animalis* subsp. *lactis, B. bifidum, B. breve, B. dentium, B. longum* subsp. *longum, B. longum* subsp. *infantis*	Genes expressed *in vitro* and *in vivo*, regulated by carbon source and GIT-related stress, enhanced *in vivo*, pili detected on different strains by atomic force microscopy, heterologous expression of *pil*2 and *pil*3 genes of *B. bifidum* PRL2010 in *L. lactis* enhance binding to ECM proteins, expression of *pil*2 increases adhesion to IECs	Foroni et al., 2011; O’Connell Motherway et al., 2011; Westermann et al., 2012; Turroni et al., 2013, 2014; Bottacini et al., 2014; Duranti et al., 2014, 2015; Wei et al., 2016
BopA	*B. bifidum*	Purified BopA inhibits and homologous and heterologous expression increases adhesion to IECs, role in adhesion recently challenged as a BopA antibody did not inhibit adhesion	Guglielmetti et al., 2008; Gleinser et al., 2012; Kainulainen et al., 2013
Transaldolase	*B. bifidum, B. longum* subsp. *Longum*	Binds to mucus, protein present on the surface of *B. bifidum* strains, protein level in *B. longum* proteome increased *in vivo*, differential expression of different isoforms in the presence of IECs	Yuan et al., 2008; González-Rodríguez et al., 2012; Wei et al., 2014
DnaK	*B. animalis* subsp. *Lactis*	Binds to plasminogen	Candela et al., 2010
Enolase	*B. bifidum, B. animalis* subsp. *lactis, B. longum* subsp. *longum*	Binds to plasminogen, in *B. longum* subsp. *longum* increased expression *in vivo* and in the presence of IECs, plasminogen binding site identified	Candela et al., 2007, 2009; Wei et al., 2014
Hydrophobicity	*Bifidobacterium* sp.	Surface hydrophobicity correlates positively with autoaggregation and adhesion to IECs	Pérez et al., 1998; Del Re et al., 2000; Pan et al., 2006

Adhesion to mucus is mostly analyzed using microtiter plate assays with immobilized mucus with quantification of adherent bacteria after metabolic labeling using radioisotopes or fluorescent dyes (He et al., 2001; Izquierdo et al., 2008; González-Rodríguez et al., 2012; Kainulainen et al., 2013). Similar assays are performed to analyze adhesion to immobilized ECM proteins (Kainulainen et al., 2013) or detection of ECM proteins bound to bacterial cells or protein extracts by specific antibodies (Candela et al., 2007, 2009, 2010).

The methods and cell lines used to determine adhesion to IECs differ largely between studies and groups. The most widely used cell lines are Caco-2, HT-29, and T84 (Guglielmetti et al., 2009; Preising et al., 2010; Gleinser et al., 2012; González-Rodríguez et al., 2012; Kainulainen et al., 2013; Grimm et al., 2014). In studies that employ more than one cell line, absolute adhesion of different strains may vary between cell lines but relative differences between strains are usually conserved (Riedel et al., 2006; Preising et al., 2010; Gleinser et al., 2012). One observation is that, although there is considerable strain-to-strain variability, strains of the species *B. bifidum* generally tend to adhere better to IECs than strains of other species (Guglielmetti et al., 2008; Gleinser et al., 2012). Detection of adherent bacteria is performed by metabolic labeling using radionucleotides (Riedel et al., 2006; Kainulainen et al., 2013), enumeration of colony forming units of adherent bacteria (Gleinser et al., 2012; González-Rodríguez et al., 2012), microscopic imaging and calculation of adhesion indices, i.e., the ratio of adherent bacteria and cells (Guglielmetti et al., 2008, 2009, 2010), or expression of fluorescent proteins (Grimm et al., 2014). However, the method of quantification does not seem to impact on adhesion itself as comparable results are obtained using radioactive or fluorescent labeling and plate counting (Riedel et al., 2006; Gleinser et al., 2012; Grimm et al., 2014).

In the following sections, the current knowledge on bifidobacterial adhesion to host structures will be summarized and the involved factors will be compared to adhesion factors of pathogens.

### Pili

A wide range of Gram-positive and -negative bacteria possess proteinaceous surface appendages termed fimbriae or pili (Proft and Baker, 2009). In general, pili are adhesive structures that are involved in biofilm formation, conjugation, motility, and adhesion to biotic and abiotic surfaces (Maier and Wong, 2015). These hair-like structures extend to some distance (up to 3 μm) from the bacterial cell surface (Proft and Baker, 2009). It is hypothesized that they are able to bridge the repulsive forces between microbial cells and biological substrates, which under physiological conditions are both negatively charged (Proft and Baker, 2009). Pili are well-known for their role as virulence factors of Gram-positive and -negative pathogens and are important for initial attachment to host tissues (Telford et al., 2006; Proft and Baker, 2009).

There is increasing evidence that bifidobacteria also encode and express pilus-like structures on their cell surface. The first report of pili in bifidobacteria was the presence of genes encoding type IVb tight adherence (Tad) pili in *B. breve* UCC2003 (O’Connell Motherway et al., 2011). Since then, genes for Tad, Type VIa, and/or sortase-dependent pili were found in basically all sequenced genomes of bifidobacteria (**Table 1**). Interestingly, in most cases bifidobacteria posses more than one pilus-coding locus and *B. dentium* harbours as much as seven gene clusters for sortase-dependent pili (Foroni et al., 2011).

Transcriptional analysis revealed that at least some of the genes are expressed under *in vitro* conditions and are regulated in response to substrate, presence of other bacteria, growth phase, or stress conditions related to the GIT (Foroni et al., 2011; Westermann et al., 2012; Duranti et al., 2014; Turroni et al., 2014). Moreover, pilin proteins are present in the *in vitro* proteome of *B. bifidum* S17 (Wei et al., 2016) and pilus-like structures were observed on several bifidobacteria by electron and atomic force microscopy (Foroni et al., 2011; O’Connell Motherway et al., 2011; Duranti et al., 2014). Collectively, this suggests that bifidobacteria possess functional pili. There is also evidence that bifidobacterial pili have a role in colonization of the host and attachment to epithelial cells. In *B. breve* UCC2003, expression of the *tad* locus was up-regulated in the GIT of mice and is required for efficient colonization in the presence of a competing microbiota (O’Connell Motherway et al., 2011). In a *B. adolescentis* strain, expression of pilus gene clusterss and presence of pili were enhanced when bacteria were isolated from the murine GIT or grown on starch, cellobiose or maltodextrin, i.e., substrates abundantly present in the GIT (Duranti et al., 2014). Similarly, expression of two of the three sortase-dependent pili clusters of *B. bifidum* PRL2010 is enhanced in the murine GIT and in the presence of human IECs *in vitro* (Turroni et al., 2013). Although a direct role for colonization and adhesion by inactivation of the corresponding genes is missing (probably due to the lack of appropriate genetic tools for the species *B. bifidum*), heterologous expression of the two gene clusters in *Lactococcus lactis* led to presence of pilus-like structures. Moreover, the recombinant *L. lactis* strains displayed increased adhesion to cultured IECs (*pil2* cluster) and ECM proteins laminin, fibronectin, fibrinogen, and plasminogen (*pil2* and *pil3* clusters; Turroni et al., 2013). Adhesion to fibronectin seems to be mediated by sugar-binding domains of the pili since enzymatic deglycosylation of fibronectin markedly reduced adhesion of the recombinant *L. lactis* strains expressing the *pil2* and *pil3* gene clusters of *B. bifidum* PRL2010 (Turroni et al., 2013).

### Moonlighting Proteins

A rather obscure group of proteins involved in adhesion of bacteria to host tissues are so-called moonlighting proteins (Huberts and van der Klei, 2010). These proteins are multifunctional and usually have an enzymatic role in bacterial metabolism or other cellular processes but at the same time are involved in totally unrelated biological functions (Huberts and van der Klei, 2010). In more than 90 pathogenic bacteria, proteins with a moonlighting function in virulence have been identified (Henderson, 2014). Interestingly, a large number of moonlighting proteins are cytoplasmic enzymes of the central metabolism that lack secretion signals raising the question if these proteins are actively exported to mediate virulence related functions. The best characterized examples are adhesins of pathogenic bacteria that are involved in primary attachment to host tissue and are important for later stages of infection (Henderson and Martin, 2011).

Enzymes of glycolysis with a moonlighting function in adhesion of pathogens include aldolase (or transaldolase), enolase, and glyceraldehyde-3-phosphate dehydrogenase (Henderson and Martin, 2011). These proteins were detected in proteomes of different bifidobacteria (Yuan et al., 2006, 2008; Ruiz et al., 2009; Gilad et al., 2011; Liu et al., 2011; Wei et al., 2016; Zhu et al., 2016). Transaldolase, a cytoplasmatic key enzyme of the bifidus shunt, was found to be present on the surface of several *B. bifidum* strains (González-Rodríguez et al., 2012). Using an *in vitro* binding assay the transaldolase could be identified as a mucin-binding protein and the specificity of this interaction was confirmed by increased mucus binding of recombinant *L. lactis* strains expressing transaldolase (González-Rodríguez et al., 2012). Enolase of different *B. longum, B. bifidum, B. animalis* subsp. *lactis* and *B. breve* strains was shown to interact with plasminogen (Candela et al., 2007, 2009; Wei et al., 2014). Moreover, the plasminogen binding site in the *B. lactis* enolase was shown to be homologous to that of *Streptococcus pneumoniae* and specific amino acid residues crucial for plasminogen binding have been identified (Candela et al., 2009). Another moonlighting protein that serves as an adhesin for bifidobacteria is DnaK, which has a primary function as a chaperone (Henderson and Martin, 2011). For *B. animalis* subsp. *lactis* BI07, DnaK was shown to bind plasminogen (Candela et al., 2007, 2010). Further potential moonlighting proteins of *B. animalis* subsp. *lactis* BI07 with plasminogen-binding activity are glutamine synthetase, bilesalt hydrolase, and phosphoglycerate mutase (Candela et al., 2007).

For *B. longum* NCC2705, transaldolase was detected at higher levels incubated *in vivo* in a rabbit intestinal loop compared to *in vitro* growth (Yuan et al., 2008) and enolase and transaldolase were more abundant in the proteome following co-cultivation with IECs (Wei et al., 2014). Also, expression of DnaK and enolase is upregulated in several bifidobacteria in response to bile (Savijoki et al., 2005; Candela et al., 2010). This indicates that bifidobacteria might be able to sense the conditions of the intestinal environment and presence of IECs (or receptors on IECs) and respond by enhancing expression of adhesive molecules.

### Other Adhesion Factors

A rather general, and non-specific property of bacteria that has been associated occasionally with adhesion of pathogens to host tissue is surface hydrophobicity (Hirt et al., 2000; Kouidhi et al., 2010). Several studies have tested different strains and species of bifidobacteria for hydrophobicity, autoaggregation and adhesion to IECs (Pérez et al., 1998; Del Re et al., 2000; Pan et al., 2006). Overall, the results suggest that (i) strains with higher surface hydrophobicity show higher autoaggregation and adhesion to IECs and (ii) *B. bifidum* strains tend to be more hydrophobic than strains of other *Bifidobacterium* sp. This is in line with other studies showing that *B. bifidum* strains adhere better to IECs than strains of other species (Preising et al., 2010; Gleinser et al., 2012).

Other non-proteinaceous component of the bacterial envelope that have been associated with adhesion to host tissue of Gram positive pathogens are glycoconjugates including exopolysaccharides, lipoteichoic, and wall teichoic acids (Weidenmaier and Peschel, 2008; Tytgat and Lebeer, 2014; Tan et al., 2015). Despite the presence of genes (potentially) involved in biosynthesis of exopolysaccharides and teichoic acids in most of the sequenced genomes of bifidobacteria (Hidalgo-Cantabrana et al., 2014; Colagiorgi et al., 2015), a contribution to adhesion have not been demonstrated conclusively so far. However, one study links exopolysaccharide production of bifidobacteria with adhesion to mucus by showing that purified exopolysaccharides of two bifidobacteria reduced adhesion of intact bacterial cells of these strains (Ruas-Madiedo et al., 2006).

Besides the abovementioned pili and moonlighting proteins no specific adhesins such as intimin, internalins, lectins, fibronectin-binding proteins as described for a number of pathogens (Niemann et al., 2004; Kline et al., 2009) have been characterized for bifidobacteria. A bioinformatic screen of the genome of *B. bifidum* S17 yielded a number of proteins with domains such as fibronectin type III domain, concanavalin A-like lectin, and collagen triple helix repeat domains, suggesting that bifidobacteria might have similar adhesins (Westermann et al., 2012). A definite role of the corresponding proteins in adhesion to host structures has yet to be demonstrated.

One specific protein that has been suspected to play a role in adhesion of bifidobacteria to IECs is BopA, lipoprotein of the cell envelope specifically found in *B. bifidum* strains (Guglielmetti et al., 2008; Gleinser et al., 2012). However, BopA contains the characteristic domains of a solute-binding protein and is part of an operon that encodes a putative oligopeptide ABC-transporter (Gleinser et al., 2012). Moreover, a recent study has challenged the idea that BopA serves a function in adhesion by showing that blocking BopA using a specific antibody does not affect adhesion of *B. bifidum* MIMBb75 to IECs (Kainulainen et al., 2013). Thus, BopA might be another example for a moonlighting protein but whether it has a role in adhesion of *B. bifidum* strains to intestinal tissue in humans needs to be elucidated in further studies.

## Conclusion

A large number of *Bifidobacterium* sp. strains were shown to adhere to IECs, mucus, and/or ECM proteins. For some bifidobacteria, adhesive structures have been characterized and include pili and different moonlighting proteins. Lactobacilli, another group of potential probiotic, Gram-positive microorganisms use exactly the same structures to adhere to the same target sites on host tissues (Vélez et al., 2007; van Tassell and Miller, 2011). Pathogenic microorganisms employ similar or even identical structures to adhere to host structures. The genus *Bacteroides* contains highly abundant commensal species as well as opportunistic pathogens that even may cause cancer (Wexler, 2007; Sears et al., 2014). Both commensal and pathogenic strains were shown to adhere to IECs, ECM, or mucus (Brook and Myhal, 1991; Ferreira et al., 2002; Macfarlane et al., 2005; de O Ferreira et al., 2006; Huang et al., 2011; Ferreira Ede et al., 2013) and pili, specific ECM-binding proteins, EPS etc. (Brook and Myhal, 1991; de O Ferreira et al., 2006; Pumbwe et al., 2006; Ferreira Ede et al., 2013) are involved in the process. Collectively, this illustrates that both pathogenic and commensal, in some cases even beneficial, bacteria employ the same strategies to attach to host structures. There is no doubt that adhesion of pathogens to host tissue is required or helps to promote infection. Bifidobacteria are generally regarded as safe microorganisms, which despite intensive studies of the past decades have not shown any pathogenic potential whatsoever. Instead, there are a number of health-related benefits associated with bifidobacteria. Although definitive proof is missing in most cases, it is reasonable to assume that adhesion to host tissue by beneficial bacteria are also required for or support their health-promoting effects. Moreover, the impressive number of different adhesion factors encoded by individual strains of bifidobacteria suggests that adhesion to host tissue is important for bifidobacteria to colonize and strive in the highly competitive ecosystem of the GIT.

## Author Contributions

All authors listed, have made substantial, direct and intellectual contribution to the work, and approved it for publication.

## Conflict of Interest Statement

The authors declare that the research was conducted in the absence of any commercial or financial relationships that could be construed as a potential conflict of interest.

## Abbreviations

ECM: extracellular matrix
GIT: gastrointestinal tract
IECs: intestinal epithelial cells
